# High Expression of HuR in Cytoplasm, but Not Nuclei, Is Associated with Malignant Aggressiveness and Prognosis in Bladder Cancer

**DOI:** 10.1371/journal.pone.0059095

**Published:** 2013-03-13

**Authors:** Yasuyoshi Miyata, Shin-ichi Watanabe, Yuji Sagara, Kensuke Mitsunari, Tomohiro Matsuo, Kojiro Ohba, Hideki Sakai

**Affiliations:** Department of Nephro-Urology, Nagasaki University Graduate School of Biomedical Sciences, Nagasaki, Japan; University of Nebraska Medical Center, United States of America

## Abstract

**Introduction:**

Human antigen R (HuR) regulates the stability of mRNA and is associated with cell proliferation, angiogenesis, and lymphangiogenesis. However, the clinical significance and pathological role of HuR in bladder cancer remains unclear. The main objective of this investigation was to clarify the relationships between HuR expression and clinical significance and cancer cell proliferation, angiogenesis, lymphangiogenesis, and expressions of cyclooxygenase (COX)-2 and vascular endothelial growth factor (VEGF)-A, -C, and -D.

**Methods:**

All expressions were examined by immunohistochemical techniques in 122 formalin-fixed specimens of bladder cancer patients. HuR expression was evaluated separately with cytoplasmic and nuclear staining. Cell proliferation, angiogenesis and lymphangiogenesis were measured as the percentage of Ki-67-positive cell (proliferation index, PI), CD34-stained vessels (microvessel density, MVD), and D2-40-stained vessels (lymph vessel density, LVD). Relationships between each HuR expression and clinicopathological features, prognosis, and expressions of COX-2 and VEGFs were analyzed by multi-variate analyses. HuR expression was also investigated in 10 mice of N-Butyl-N-[4-hydroxybutil] nitrosamine (BBN) induced bladder cancer model.

**Results:**

In human tissues, high cytoplasmic expression was seen in 5% and 25.4% of normal and cancer cells, respectively. Nuclear HuR expression bore no significant relationship to any pathological features. However, cytoplasmic HuR expression appeared positively associated with pT stage and grade (*P*<0.001). In mouse tissues, similar trends were confirmed. Cytoplasmic expression correlated with PI, MVD, and LVD, as well as expression of VEGF-A and -C, but not VEGF-D. High cytoplasmic expression of HuR was a significant predictor of metastasis and cause-specific survival, and was identified as a prognostic correlative factor for metastasis (hazard ratio, 4.75; P = 0.028) in a multivariate analysis model that included pathological features.

**Conclusions:**

Cytoplasmic HuR appears to play important roles in cell proliferation, progression, and survival of bladder cancer patients. Its expression was associated with angiogenesis, lymphangiogenesis, and expressions of VEGF-A and –C.

## Introduction

Regulation of mRNA decay is an essential mechanism for controlling gene expression. The control of mRNA stability depends on sequences in the transcript itself and on RNA-binding proteins that dynamically bind to these sequences. Human antigen R (HuR) is a member of the embryonic-lethal, abnormal vision (ELAV)-like protein family of RNA-binding proteins, and is reported to be a multifunctional protein that has been implicated in the regulation of various aspects of RNA metabolism. That is, HuR is involved in post-transcriptional regulation of the turnover and stability of RNA [1].

HuR is also reported to regulate cell proliferation and tumor-associated inflammation [2], [3]. In addition, HuR has been found to be involved in the regulation of angiogenesis by interacting with the transcripts of numerous angiogenesis-promoting factors, and is also known to correlate with lymphangiogenesis in a variety of pathological conditions, including cancer [3], [4]. Based on these facts, it has been suggested that HuR plays important roles in carcinogenesis, tumor growth, metastasis, and prognosis in malignancies. Actually, several reports have described HuR expression showing positive associations with malignant aggressiveness and serving as a prognostic factor for poor clinical outcome in various cancers [5]–[7]. On the other hand, markedly different findings have been reported in patients with breast cancer [8]. The pathological roles and prognostic value of HuR expression in cancer patients thus remain contentious, and the clinical significance and predictive value for prognosis in patients with bladder cancer has yet to be clarified.

As mechanisms for these pathological activities, vascular endothelial growth factors (VEGFs) and cyclooxygenase (COX)-2 are the most well-known HuR-associated factors [5], [7], [9]–[11]. These proteins have also been reported to be associated with malignant aggressiveness and prognosis in patients with bladder cancer [12], [13]. However, little information has been accumulated regarding the relationship between HuR expression, VEGF-A, -C, and -D, and COX-2 in human bladder cancer tissues.

The intracellular location of HuR has been reported to be predominantly nuclear in many types of cancer cells [14], [15]. However, HuR shuttles from the nucleus to cytoplasm in response to various stimuli, prolonging mRNA half-life and facilitating the efficient translation of proteins [15]. Given these facts, investigation as to the clinical, pathological, and prognostic roles of nuclear and cytoplasmic expression of HuR is warranted.

The main purpose of this study was to clarify the pathological significance and prognostic value of nuclear and cytoplasmic HuR expression in patients with bladder cancer. To confirm the pathological significance of HuR under a given tissue microenvironment, chemically induced bladder cancer mouse model was used for additional experiments. In addition, relationships between these HuR expressions, angiogenesis and lymphangiogenesis, and VEGF-A, -C, -D, and COX-2 expressions were also examined in patients with bladder cancer.

## Materials and Methods

### Ethics Approval

The study was conducted according to the Helsinki II declaration and it was approved by the Ethics Review Committee of the Nagasaki University Hospital, Nagasaki, Japan.

Written informed consent was obtained from the patients involved in our study before their enrollment. Animals in this study were handled according to the Guidelines for Animal Experiments of Nagasaki University, and the Regulations of Animal Care and Use Committee approved the study protocol.

### Patients

All slides of 122 bladder cancer specimens obtained from transurethral resection (TUR) at our hospital between 1993 and 2004 were reviewed. All patients had been clinically diagnosed with non-muscle invasive bladder cancer (NMIBC) without metastasis. We excluded patients who had received neoadjuvant therapy. In addition, specimens that cancer cell number was under 500 were also excluded from this study. On the other hand, among the 122 patients, adjuvant therapy including systematic chemotherapy, intravesical chemotherapy, and intravesical bacillus-Calmette-Guérin were performed in 14 (11.5%), 67 (54.9%), and 19 patients (15.6%), respectively. All patients were evaluated by chest radiography, ultrasonography, and computed tomography (CT) of the urinary bladder and abdomen, and cystoscopy. In addition, CT of the lung or brain, magnetic resonance imaging (MRI), drip-infusion pyelography, and bone scans were performed as deemed necessary. Tumors were staged according to the American Joint Committee on Cancer and graded according to the World Health Organization and International Society for Urological Pathology classification system. In the present study, tumors were grouped for statistical analysis into the following groups: low- (pTa+1) and high-stage (≥T2); or low- (grades 1 and 2) and high-grade (grade 3). We also examined 20 tissue samples of normal urinary bladder obtained from apparently normal areas of the bladder of patients with transitional cell carcinoma of the upper urinary tract. All of control patients showed G1 and non-muscle invasive disease, and they showed recurrence within a follow-up period of 10–17 years. The median duration of follow-up was 51 months (range, 2–182 months).

### Animal

HuR expression was examined in 10 samples of normal urothelial cells (without BBN), 5 of NMIBC (BBN solution for 14 weeks), and 5 of muscle invasive bladder cancer (MIBC) (for 24 weeks) of chemical induced bladder cancer mouse model. This model was used in a previous report [16].

### Immunohistochemistry

Immunohistochemical examinations were performed using formalin-fixed, paraffin-embedded sections. We used anti-HuR antibody (Santa Cruz Biotechnology, Santa Cruz, CA) as the primary antibody (rabbit polyclonal, reactive for both of human and mouse HuR). Five-micrometer-thick sections were deparaffinized in xylene and rehydrated in graded solutions of ethanol. Antigen retrieval was performed at 100°C for 15 min. in 0.01 M sodium citrate buffer (pH 6.0). All sections were then immersed in 3% hydrogen peroxide for 30 min to block endogenous peroxidase activity. Sections were incubated overnight with the primary antibody at 4°C, then washed in 0.05% Tween 20 in phosphate-buffered saline (PBS). Next, sections were incubated with peroxidase using the labeled polymer method with Dako EnVision+™ Peroxidase, (Dako, Carpinteria, CA) for 60 min. The peroxidase reaction was visualized with the liquid 3,3'-diaminobenzidine tetrahydrochloride (DAB) substrate kit (Invitrogen Corporation, Carlsbad, CA). Sections were counterstained using hematoxylin. Other methods were performed as described previously [13], [17], [18]. Briefly, we also evaluated VEGF-A, -C, and -D and COX-2 expressions in similar specimens using immunohistochemical techniques. In addition, microvessel density (MVD) and lymph vessel density (LVD) were estimated using CD34-positive lumina and D2-40-positive lumina, respectively. Details of the methods for determining MVD and LVD were as described previously [13]. We performed all evaluation anew for this study. A variety of cancer specimens that had been confirmed in preliminary studies as immunoreactive for the studied antigens were used as positive controls for HuR (liver), VEGFs (renal cell carcinoma), COX-2 (colon), D2-40 (tonsil), and CD34 (kidney). The specificities of these specimens as positive controls were confirmed in our previous reports [13], [17], [18]. A consecutive section from each sample processed without the primary antibody was used as a negative control. Positive and negative controls were set up for each batch of experiments. In addition, to confirm the specificity of HuR immunoreactivity, we performed similar investigation by using goat polyclonal antibody (Santa Cruz Biotechnology, Santa Cruz, CA) in samples of human (n = 40) and mouse tissues (n = 20), and they were also incubated with blocking peptide for this anti-HuR antibody (Santa Cruz Biotechnology, Santa Cruz, CA).

### Evaluation

HuR expression was evaluated by immunoreactive score as reported previously [19]. Briefly, cytoplasmic and nuclear staining patterns were scored using the following scales: 0, no staining; 1, weak and/or focal staining (<10% of cells); 2, moderate or strong staining (10–50% of cells); and 3, moderate or strong staining (>50% of cells). Scores 0 and 1 were judged as low expression, and scores 2 and 3 were judged as high expression. This evaluation method was used for mouse tissues. With regard to other expressions, results were considered positive if staining intensity was strong, and the percentage of positively stained cancer cells was determined using a continuous scale according to previous reports [13], [17]. Briefly, expression levels were assessed semi-quantitatively from the percentage of expressing carcinoma cells (from ≥500 carcinoma cells). These cells were examined using an E-400 microscope (Nikon, Tokyo, Japan) and digital images were captured (DU100; Nikon). In addition, we used a computer-aided image analysis system (Win ROOF version 5.0; Mitani, Fukui, Japan) to calculate statistical variables. Two investigators (S.W. and Y.M.), blinded to clinical features and survival data, independently performed semi-quantitative analyses and immunostaining interpretations. The rate of disagreement in analysis between these two investigators was <10% and the mean density was used for statistical analyses.

### Statistical Analysis

Data are expressed as means (standard deviation [SD]). Student’s t-test was used for analysis of continuous variables. The chi-square test and Fisher’s exact test were used for comparisons of categorical data. Spearman’s correlation coefficient was used to determine associations between two continuous variables. Crude and adjusted effects were estimated by logistic regression analysis and described as odds ratios (OR) with 95% confidence intervals (95%CIs), together with *P*-values. Survival analysis was evaluated using Kaplan-Meier analysis and the log-rank test, and variables that achieved statistical significance (*P*<0.050) in univariate analyses were subsequently entered into a multivariate analysis using Cox proportional hazards analysis (described as hazard ratio [HR] with 95%CI, together with the *P*-values) (Model A). In addition, to examine the predictive value of HuR expression in greater detail, the analysis included a multivariate model including all risk factors (Model B). All statistical tests were two-sided and significance was defined as *P*<0.050. All statistical analyses were performed on a personal computer with StatView for Windows version 5.0 software (Abacus Concepts, Berkeley, CA).

## Results

### Localization and Expression of HuR

**Figure 1:**
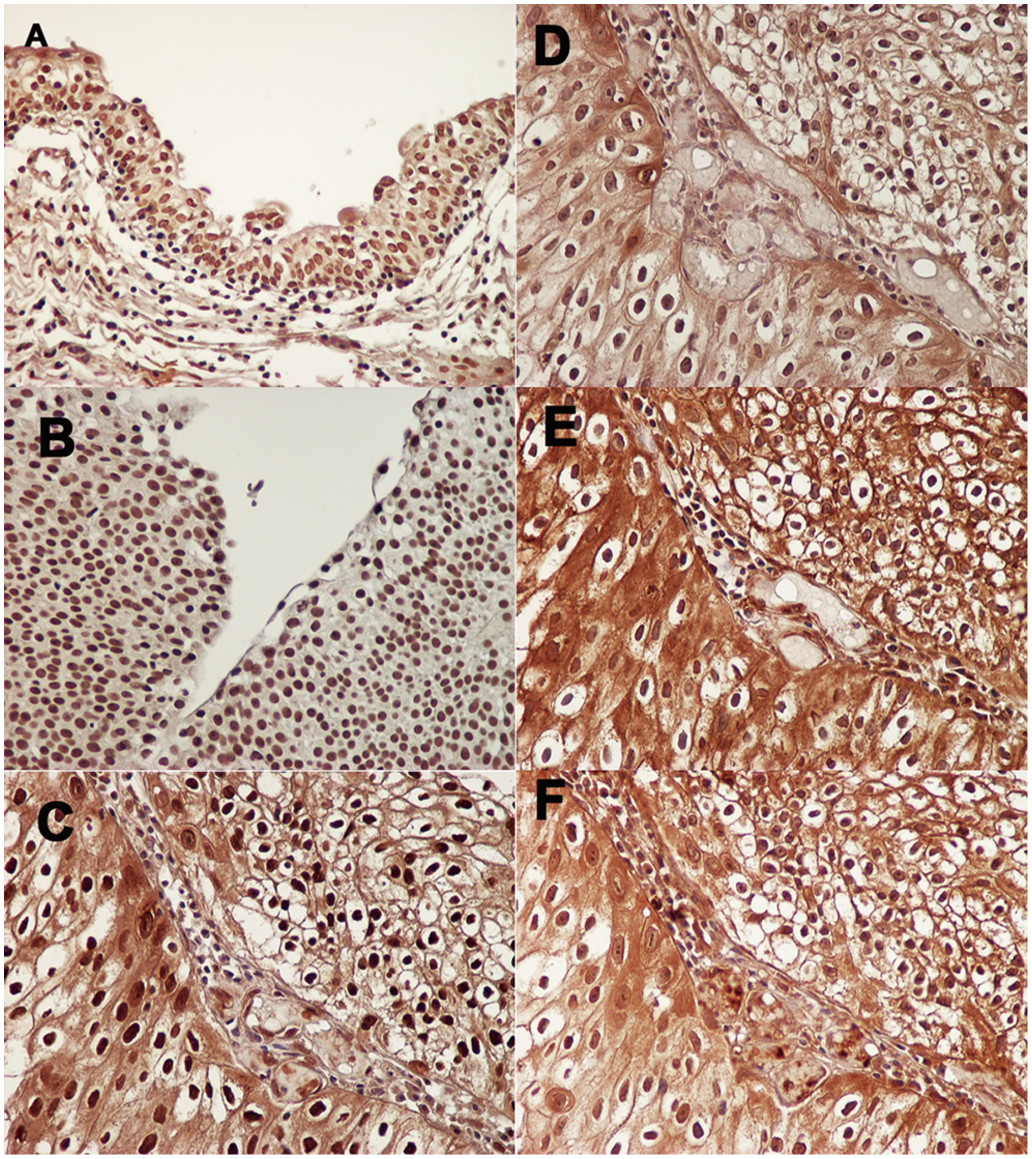
Representative examples of HuR immunoreactivity in normal urothelial cells (A; magnification, ×200.) and cancer cells (B and C; magnification, ×400.). HuR was mainly detected in nuclei of both normal and cancer cells in bladder tissues. Moderate or strong cytoplasmic expression was evident in cancer cells (C), but was rare in normal urothelial cells (A). Some cancer cells are also showed weak cytoplasmic expression although nuclear expression was detected (B). In addition, we showed representative figures of HuR expression (C) and factors related thereto; (D) cyclooxygenase-2, (E) vascular endothelial growth factor-A, (F) vascular endothelial growth factor-C in same area. (all magnification, x400).

In normal urothelial cells, weak to moderate nuclear HuR expression was detected, along with absent to weak cytoplasmic expression (Fig. 1A). Finally, 90% (18/20) was judged as showing high nuclear expression in normal urothelial cells. In contrast, high cytoplasmic expression was seen in only 5% (1/20) of normal tissues. With regard to nuclear HuR expression in cancer cells, expression was high in 88 specimens (72.1%; score 2, n = 64; score 3, n = 24) and low in 34 (27.9%; score 0, n = 1; score 1, n = 33). On the other hand, with regard to cytoplasmic expression in cancer cells, expression was high in 31 specimens (25.4%; score 2, n = 24; score 3, n = 7) and low in 91 (74.6%; score 0, n = 37; score 1, n = 54). Representative examples of low and high HuR expression in cancer tissue are shown in Figure 1B and C, respectively.

**Figure 2:**
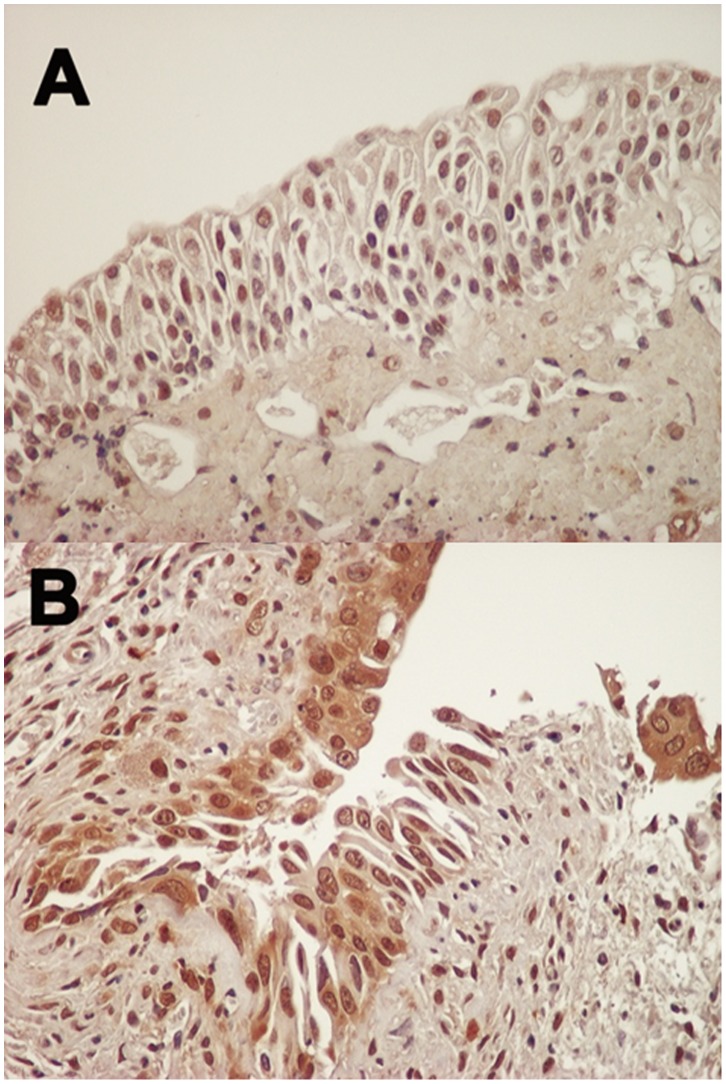
Representative examples of HuR immunoreactivity in normal cells (A) and cancer cells (B) of chemical-induced bladder cancer mouse model. (Magnification, x400). Staining pattern was close to human tissues.

In animal experiments, high nuclear expression was found in 80% (8/10) and 70% (7/10) of normal urothelial and cancer cells, respectively. On the other hand, high cytoplasmic expression in normal cells was detected in 10% (1/10). In cancer cells, high expression in NMIBC and MIBC was detected in 40% (2/5) and 80% (4/5), respectively. Representative examples of HuR expression in mice tissues were showed in Figure 2A and B. Importantly, similar results were obtained using another anti-HuR antibody and its staining was reduced by competition with blocking peptide.

### Clinical and Pathological Significance of HuR Expression

Relationships between pathological features and HuR expression in the nucleus and cytoplasm are shown in Table 1. Nuclear HuR expression showed no significant relationship to pT stage or grade (*P* = 0.471 and *P* = 0.773, respectively). Conversely, cytoplasmic HuR expression was positively associated with pT stage (*P*<0.001) and grade (*P*<0.001).

**Table 1:** Relationships between pathological features and HuR expression.

		HuR in nucleus (%)		HuR in cytoplasm (%)	
	N	Negative	Positive	Negative	Positive
**pT stage**					
**Ta**	33	12 (36.4)	21 (63.6)	31 (93.9)	2 (6.1)
**T1**	70	18 (25.7)	52 (74.3)	57 (81.4)	13 (18.6)
**T2**	11	2 (18.2)	9 (81.8)	2 (18.2)	9 (81.8)
**T3**	8	2 (18.2)	6 (75.0)	1 (12.5)	7 (87.5)
**Low (Ta+1)**	103	30 (29.1)	73 (70.9)	88 (85.4)	16 (14.6)
**High(T2+3)**	19	4 (21.1)	15 (78.9)	3 (15.8)	16 (84.1)
***P*** ** value**		0.471		<0.001	
**Grade (G)**					
**G1**	34	10 (29.4)	24 (70.6)	28 (82.4)	6 (17.6)
**G2**	43	10 (29.4)	33 (76.4)	38 (88.3)	5 (11.7)
**G3**	45	14 (31.1)	31 (68.9)	25 (55.6)	20 (44.4)
**Low (G1+2)**	77	20 (25.9)	57 (74.1)	66 (85.7)	11 (14.3)
**High (G3)**	45	14 (31.1)	31 (68.9)	25 (55.6)	20 (44.4)
***P*** ** value**		0.773		<0.001	

As shown in Table 2, cytoplasmic HuR expression was positively associated with expressions of COX-2 (*P* = 0.011), VEGF-A (*P* = 0.021) and VEGF-C (*P* = 0.004), but not with VEGF-D expression (*P* = 0.134). In addition, cytoplasmic HuR expression also displayed positive correlations with MVD (*P* = 0.010) and LVD (*P* = 0.023). On the other hand, nuclear expression of HuR showed no significant correlation with any of these expressions or variables (Table 2). Thus, representative examples for these significant molecules are shown in the high HuR expression sample (Figure 1D–F).

**Table 2:** Correlation with MVD, LVD and expressions of COX-2 and VEGF family.

	HuR in nucleus			HuR in cytoplasm		
	Negative	Positive	*P value*	Negative	Positive	*P Value*
**COX-2** **(%)**	21.6 (8.5)	20.9 (9.3)	0.690	19.8 (8.9)	24.6 (8.7)	0.010
**VEGF-A**	33.6 (14.7)	33.9 (12.5)	0.919	32.2 (12.9)	38.5 (12.9)	0.021
**VEGF-C**	30.8 (15.5)	31.7 (16.3)	0.792	29.0 (14.6)	38.6 (17.8)	0.004
**VEGF-D**	31.0 (16.5)	32.1 (15.3)	0.737	30.6 (15.0)	35.4 (16.7)	0.134
**MVD** **(/mm^2^)**	69.9 (19.7)	67.8 (18.2)	0.599	65.9 (18.7)	75.8 (16.3)	0.010
**LVD** **(/mm^2^)**	25.1 (9.5)	27.8 (12.9)	0.271	25.6 (11.1)	31.1 (13.8)	0.023

COX: cyclooxygenase, VEGF: vascular endothelial growth factor, MVD: microvessel density, LVD: lymph-vessel density.

### Relationships between HuR-related Molecules and Angiogenesis or Lymphangiogenesis

**Figure 3:**
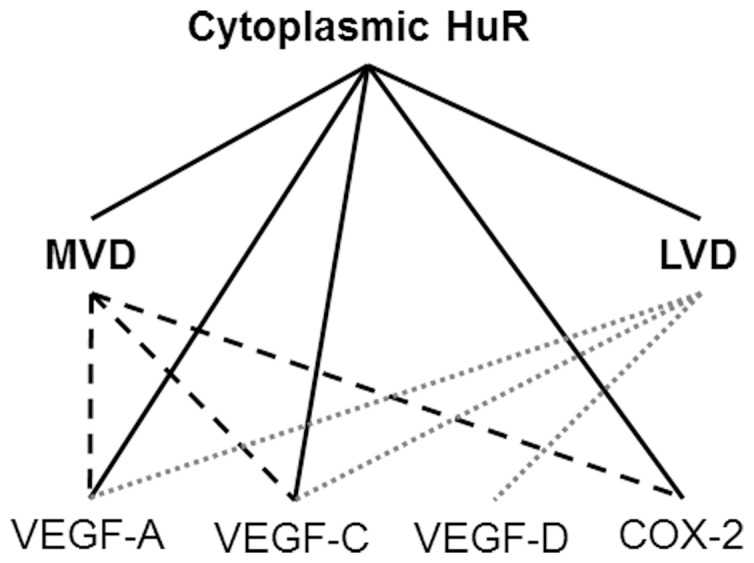
Schema of the relationships between cytoplasmic HuR expression and angiogenesis, lymphangiogenesis, and expressions of related molecules. MVD, microvessel density; LVD, lymphvessel density; VEGF, vascular endothelial growth factor; COX, cyclooxygenase.

In our study population, MVD was closely associated with VEGF-A (r = 0.40, *P*<0.001), VEGF-C (r = 0.41, *P*<0.001), and COX-2 expression (r = 0.31, *P*<0.001), but not with VEGF-D (r = 0.17, P = 0.652). Similarly, LVD was also associated with VEGF-A (r = 0.39, *P*<0.001), VEGF-C (r = 0.58, *P*<0.001), and VEGF-D (r = 0.53, P<0.001), but not with COX-2 expression (r = 0.15, *P* = 0.103). The schema of such correlations was showen in Figure 3.

To clarify the significance of expressions of VEGF-A, VEGF-C, and COX-2 for MVD and LVD in greater detail, multivariate analysis was performed with models including these factors, pT stage, and grade. With regard to MVD, although VEGF-C and COX-2 were associated with MVD (OR = 1.05, 95%CI = 1.02–1.08, *P* = 0.002 and OR = 1.05, 95%CI = 1.00–1.11, *P* = 0.043, respectively), VEGF-A expression showed the strongest association (OR = 1.05, 95%CI = 1.03–1.11, *P*<0.001). Similar analysis showed that VEGF-C was most closely associated with LVD (OR = 1.05, 95%CI = 1.01–1.08, *P* = 0.001), while VEGF-A expression was also associated (OR = 1.05, 95%CI = 1.01–1.08, *P* = 0.011).

### Survival Analyses

**Figure 4:**
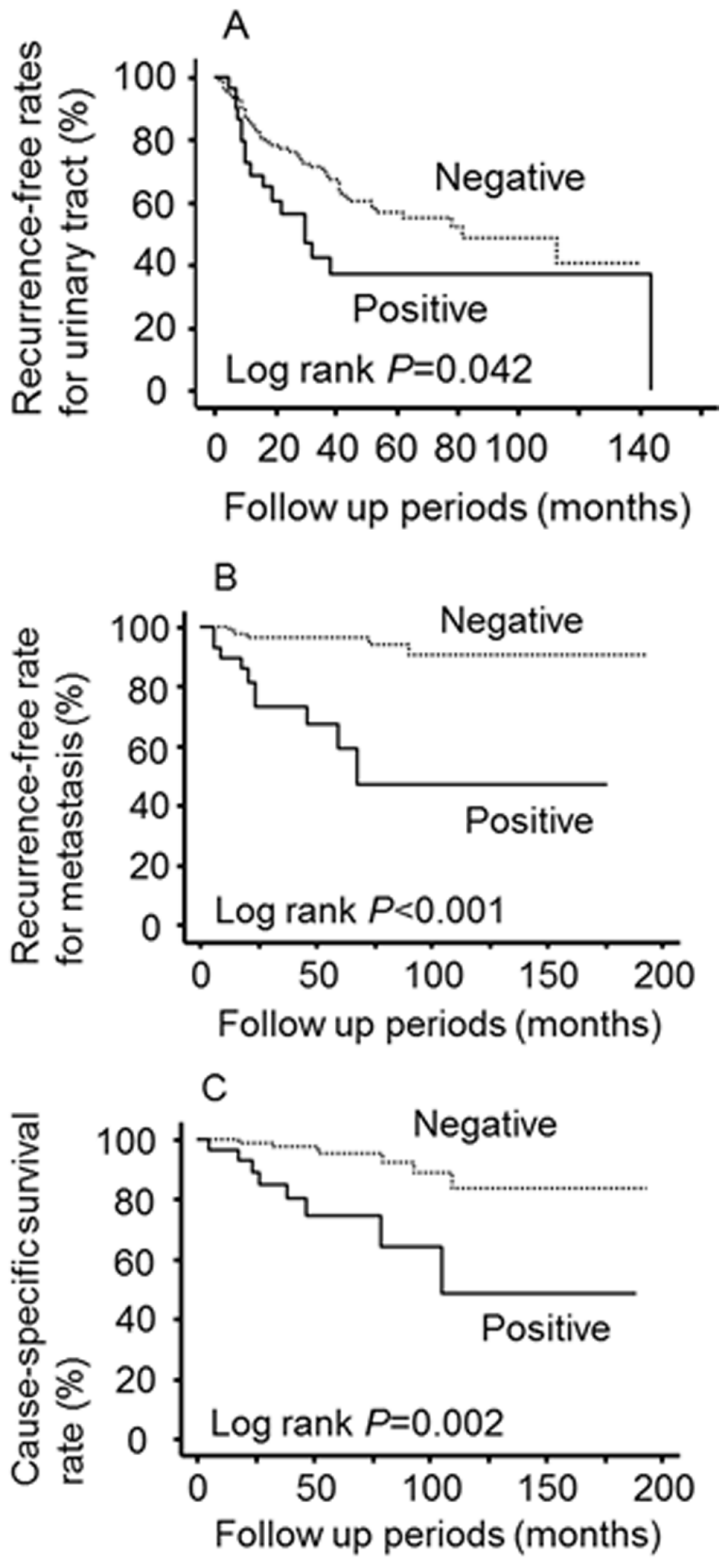
Kaplan-Meier curves for recurrence-free survival rates with urinary tract cancer (A), recurrence-free survival rates with metastasis (B), and cause-specific survival rates (C).

Nuclear HuR expression was not identified as a significant predictor of recurrence in the urinary tract, metastasis, or cause-specific survival (log-rank *P* = 0.156, *P* = 0.058, and *P* = 0.940, respectively). Conversely, high cytoplasmic HuR expression was a significant predictor for each of these parameters (Fig. 4 A–C). To show more detailed values of predictive factors, similar analyses were also performed for pathological features and adjuvant therapy. In addition, we showed multivariate analysis models including all these factors (Model A) and cytoplasmic HuR expression and pathological features (Model B) are showed in Table 3. In both models, HuR expression was identified as a prognostic factor for metastasis, but not for recurrence into the urinary tract (HR = 2.00, 95%CI = 0.98–4.11, *P* = 0.057 in Model A and HR = 1.82, 95%CI = 0.90–3.72, *P* = 0.094 in Model B) and cause-specific survival (HR = 5.42, 95%CI = 0.29–6.71, *P* = 0.688 in Model A and HR = 1.26, 95%CI = 0.42–3.75, *P* = 0.683 in Model B).

**Table 3:** Survival analyses for metastasis.

	Univariate analyses			Multivariate analyses		
	HR	95% CI	*P value*	HR	95% CI	*P value*
**Grade**						
**High** **(Model A)**	2.70	1.56–4.66	<0.001	1.47	0.49–4.40	0.489
**(Model B)**	–	–	–	1.15	0.38–3.51	0.805
**pT stage**						
**High (A)**	10.44	3.58–30.46	0.001	4.72	1.25–17.83	0.022
**(B)**	–	–	–	3.25	0.87–12.06	0.079
**Adjuvant Tx**						
**Absence (A)**	0.402	0.05–3.09	0.381	0.18	0.02–1.50	0.116
**(B)**	–	–	–	–	–	–
**HuR in cytoplasm**						
**Positive (A)**	9.60	3.19–28.88	<0.001	4.75	1.78–12.75	0.028
**(B)**	–	–	–	5.22	1.34–20.35	0.017

Tx: therapy, HR: hazard ratio, CI: confidential interval.

Model A: including cytoplasmic HuR expression, grade, pT stage, and adjuvant therapy.

Model B: including cytoplasmic HUR expression, grade, and pT stage, but not adjuvant TX.

## Discussion

The present study demonstrated that cytoplasmic HuR expression was closely associated with malignant potential, tumor progression, and outcome for bladder cancer patients. Conversely, no such significance was found for nuclear expression of HuR. Previous studies have detected cytoplasmic accumulation in several cancers, showing that nucleocytoplasmic translocation of HuR was essential for RNA stability [20], [21]. Our results support a similar role in human bladder cancer tissues.

This is the first report on relationships between HuR expression and pathological features, recurrence, and survival in patients with urothelial carcinoma of the urinary bladder cancer. With regard to the pathological significance and prognostic roles of HuR, several previous studies have reported that over-expression is associated with high-grade malignancy, advanced stage, and poor survival in patients with colon cancer [6], breast cancer [5], and renal cell carcinoma [7]. However, HuR expression does not appear to be associated with pathological status in patients with breast cancer, with high expression of HuR predicting a better prognosis [8]. Our results support former situation in patients with bladder cancer. As a mechanism underlying such pathological activities of HuR, regulation of angiogenesis has been suggested in various cancers [3], [4]. Special attention was paid to the relationships between HuR expression, malignant potential and pathological features because stimulation of angiogenesis has been linked to tumor growth and progression in patients with several malignancies, including bladder cancer [13], [22]. Finally, Our results showed the possibility that cytoplasmic expression of HuR was positively associated with malignant behavior and outcome in bladder cancer patients. In addition, there is a possibility that such malignant aggressiveness is associated with HuR-related angiogenesis.

One of the most interesting findings in this study was that cytoplasmic HuR expression correlated positively with lymphangiogenesis in bladder cancer tissues. To the best of our knowledge, this study is the first to report a relationship between HuR expression and lymphangiogenesis in patients with bladder cancer, although similar findings have been detected in lung cancer in previous reports [4]. In addition, interestingly, both that report and the present study showed that cytoplasmic HuR expression correlated with both angiogenesis and lymphangiogenesis. Given these results, we hypothesized that co-factors that can influence both angiogenesis and lymphangiogenesis may associate with such finding in cancer tissues.

Detailed regulatory mechanisms of angiogenesis and lymphangiogenesis by HuR in human cancer tissues are still not fully understood. Several molecules have been reported to be HuR-related, such as VEGF-A [10], VEGF-C, and COX-2 [7], [9]. These factors are known to be associated with MVD and LVD in bladder cancer. However, the relationships between HuR expression and these angiogenesis- and lymphangiogenesis-related molecules in human bladder cancer tissues are still unclear. Our results showed that cytoplasmic HuR expression correlated positively with expressions of VEGF-A and -C, but not VEGF-D. We have previously reported that VEGF-A and -C were significantly associated with MVD and LVD in human bladder cancer tissues [13]. In addition, such significant relationships were not detected for nuclear HuR expression. Given these findings, we speculated that translocation from nucleus to cytoplasm is an important process in stimulating angiogenesis and lymphangiogenesis. Furthermore, up-regulation of VEGF-A and -C expressions may be associated with such phenomenon in bladder cancer.

An additional important result in the present study was the fact that cytoplasmic HuR expression was a useful predictor of prognosis in bladder cancer patients who underwent TUR. In particular, expression was closely associated with postoperative metastasis. Many investigators have identified increased MVD and LVD as strong predictors of outcome in bladder cancer patients [13], [23], [24]. Based on these findings, we thought that such prognostic roles of MVD and LVD were reasonable. On the other hand, cytoplasmic expression of HuR was identified as a significant predictor of cause-specific survival in univariate analysis, but not as an independent predictor in multivariate analysis. We are not sure why such differences were encountered in this study. However, postoperative survival was influenced by numerous factors. We thus speculated that cytoplasmic HuR may play various roles in patient survival, while various other factors and molecules may have stronger effects in determining outcomes for patients with bladder cancer.

In this study, we showed that cytoplasmic HuR expressions in bladder cancer cells were higher than those in normal epithelial cells. Biological functions of HuR in human and mouse have been reported to be similar in previous report [25]. However, validation of specificity of HuR target is not still completely clear. Furthermore, newly and detailed pathological function of HuR in cancer cells are becoming more clearly in recent years [26]. Thus, further studies regarding molecular mechanism and cell biology experiments are necessary to understand more detailed pathological roles of HuR in bladder cancer.

In conclusion, our results showed that cytoplasmic HuR expression was significantly associated with malignant aggressiveness and bladder cancer patient outcomes, whereas nuclear HUR expression was not. In addition, cytoplasmic HuR expression is associated with angiogenesis, lymphangiogenesis, and these-related molecules including VEGF-A, VEGF-C, and COX-2. Our results suggest that cytoplasmic HuR expression was useful as a predictive marker for metastasis after TUR in patients with bladder cancer. To obtain further mechanistic insight, further *in vivo* and *in vitro* studies in bladder cancer are necessary.
